# COVID‐19 vaccine causing Guillain‐Barre syndrome, a rare potential side effect

**DOI:** 10.1002/ccr3.4756

**Published:** 2021-08-30

**Authors:** Ahmad S. Matarneh, Alia Hani Al‐battah, Khalid Farooqui, Mohamed Ghamoodi, Mohammed Alhatou

**Affiliations:** ^1^ Internal Medicine Department Hamad Medical Corporation Doha Qatar; ^2^ Department of Neurology Hamad Medical Corporation Doha Qatar

**Keywords:** COVID‐19 vaccine, Gullain‐Barre syndrome, neurology, pandemic

## Abstract

Patients with neurological symptoms should be enquired about recent vaccination history. It is important after the COVID‐19 mRNA vaccine, which is newly introduced as it might link to the development of a wider variety of neurological diseases.

## Graphical abstract

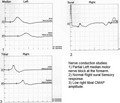

## 1 INTRODUCTION

COVID‐19 has affected the world in many ways, resulting in several challenges. First, it has resulted in high rates of morbidity and mortality. Efforts have been made to study the nature of the disease and discover a new treatment to help decrease those rates. COVID‐19 vaccines were introduced recently with a variable degree of immunogenicity and safety.

We report a 61‐year‐old man who developed Guillain‐Barre syndrome (GBS) within four days of receiving the Moderna SARS‐CoV‐2 vaccine. The patient presented with a 3‐day history of distal more than proximal, upper extremity progressive weakness following receiving the second dose mRNA‐based vaccine. Common infectious triggers of (GBS) were ruled out. His clinical picture, CSF analysis, and electrodiagnostic testing were consistent with an acute demyelinating polyneuropathy. His clinical condition significantly improved after five days course of intravenous immunoglobulin (IVIG).

COVID‐19 vaccines have been tested in large, randomized controlled trials. The vaccines have shown a high level of efficacy and safety across all populations. However, they can result in different side effects, as is the case with all other vaccines. Neurological side effects can occur following COVID‐19 vaccines. However, their frequency is not well studied yet. One of those side effects is the development of Guillain‐Barre syndrome. The frequency of its occurrence is still not precise, and many other studies are needed to clarify its incidence further. The temporal relationship between COVID‐19 vaccination and GBS development, in this case, was suggestive of a vaccine‐induced cause, and the clinical implications of this association warrant further research.

The severe acute respiratory syndrome coronavirus 2 (SARS‐CoV‐2) is a newly emerging disease entity first reported in Wuhan, China, in December 2019. It led to high morbidity and mortality rates, affecting the world's social, psychological, and financial aspects.^1^ Its presentations are widely variable depending on the system involved. The main presenting symptoms are respiratory, ranging from asymptomatic to a more severe lower respiratory tract infection, acute respiratory distress syndrome (ARDS), and in more severe cases, death. However, it can also infect the central nervous, cardiovascular, and gastrointestinal systems.^2^ Numerous treatments have been utilized for treating it, and the evidence on how to treat it is growing daily.^3^ Since its emergence, efforts have been put to find a vaccine that would halt the progression of the pandemic. In December 2020, the first COVID‐19 vaccines were successfully announced with promising results and were approved for use after an emergency authorization by FDA.^4^ Side effects secondary to these vaccines were variable, and further studies are needed to establish their safety. Some of the side effects described are myalgia, fever, and headache. Other side effects were reactivation of the COVID‐19 and ARDS.^5^ Neurological side effects are rare but have been described.^6^ We hereby report a case that we encountered after an mRNA vaccine and found features of acute demyelinating polyneuropathy as found by nerve conduction studies.

## 2 CASE PRESENTATION

A 61‐year‐old gentleman presented to the emergency department with a 3‐day history of bilateral upper limb weakness, more distally, involving the hands and described as an inability to perform fine hand movements such as buttoning his shirt and holding the pen properly when writing. He denied having any numbness or pain. There were no other neurological deficits. There was no antecedent history of respiratory tract or gastrointestinal tract infection. He had no record of a previous similar attack. His symptoms started four days after receiving the second dose of the COVID‐19 vaccine (Moderna).

On examination, the patient had normal vital signs. Neurological examination was remarkable for decreased power in the distal muscle group with a power of 4/5. Both upper limbs showed no fasciculation, no abnormal movements, and normal tone. He had normal deep tendon reflexes all over. The sensory and cerebellar examinations were normal. Neurological examination of the lower limbs was normal. Cardiac, respiratory, and abdominal examinations were normal. Complete blood count, urea, creatinine, electrolytes, C‐reactive protein, procalcitonin, vitamin B12, folic acid, and thyroid function tests were normal, which excluded an infectious or nutritional deficiency as a cause of his symptoms (Table 1). Lumbar puncture was performed, CSF analysis revealed pink fluid, RBC 3000/ul, WBC 4/ul, Glucose 4.27 mmol/L, protein 0.5 gm/L [normal range 0.15–0.45 gm/L] indicating albumin‐cytologic dissociation (Table 2).

**TABLE 1:** Basic laboratory investigations

Laboratory	Result	Reference range
WBC	7.2 × 10^6^/ul	4.5–5.5 × 10^6^/ul
Hgb	12 gm/dl	13–17 gm/dl
Urea	4.6 mmol/L	2.8–8.1 mmol/L
Creatinine	86 umol/L	62–106 umol/L
Sodium	138.0 mmol/L	136–145 mmol/L
Potassium	3.70 mmol/L	3.5–5.1 mmol/L
Calcium	2.34 mmol/L	2.15–2.50 mmol/L
AST	20.0 U/L	0–40 U/L
ALT	16.0 U/L	0–41 U/L
ALP	62.0 U/L	40–129 U/L
CRP	2.8 mg/dl	0.0–5.0 mg/dl
Lactic acid	1.0 mmol/L	0.5–2.2 mmol/L
Vitamin B12	156.0 pmol/L	145–596 pmol/L

**TABLE 2:** CSF analysis

Color	Colorless	
White blood cells	4/ul	0–5/ul
Red blood cells	3000/ul	0–2/ul
Neutrophils	75%	40%−60%
Lymphocytes	95%	40–80%
Monocytes	2%	15–45%
Glucose	4.27 mmol/L	2.22–03.89 mmol/L
Proteins	0.5 gm/L	0.15–0. 45 gm/L
AFB smear	Negative	
Oligoclonal bands	Negative	
Viral panel	Negative	
Culture	Negative	

**FIGURE 1:**
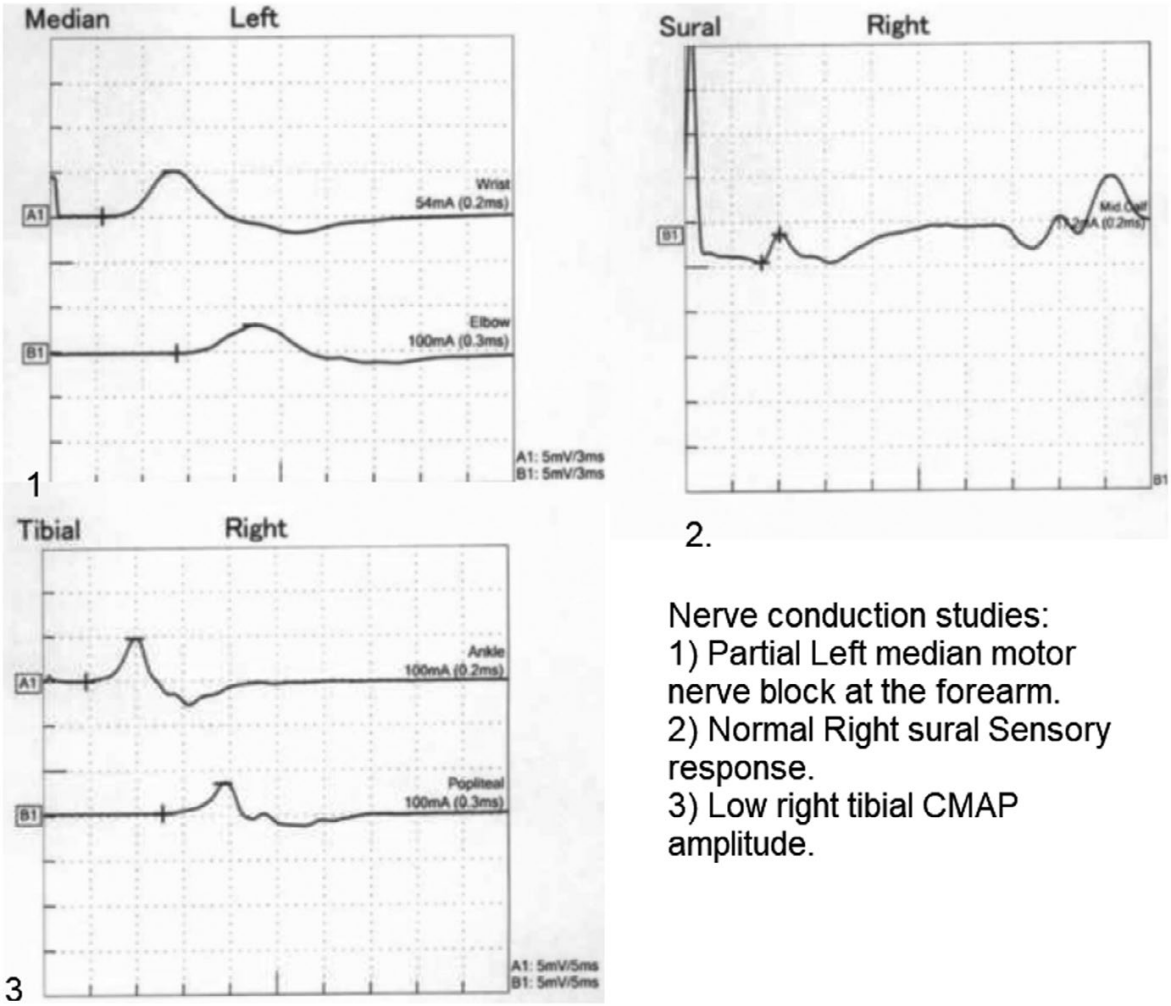
Nerve conduction studies

Nerve conduction studies showed electrophysiological evidence of pure motor neuropathy with primary demyelinating features suggestive of demyelinating polyneuropathy (Figure 1). The patient received IV immunoglobulin therapy total of 2 gm/kg; IVIG 0.4 gm/kg per day for five days. His power improved throughout his hospital stay, and he was discharged home. Upon follow‐up in the outpatient department, he had recovered entirely with full power upon examination.

## 3 DISCUSSION

COVID‐19 has affected the world in many ways. It created a high burden on countries and populations and currently remains a challenge associated with increased morbidity and mortality rates. The impact of the pandemic resulted in a considerable change in day‐to‐day life.^7^ Since Its emergence, researchers have been working robustly to find a cure. COVID‐19 vaccines were introduced recently after extensive work to find a method to halt the progression of this pandemic. New technology was introduced, which is the development of mRNA‐based vaccines; BNT162B2 and mRNA‐1273 being the primary examples. mRNA vaccines work by inducing a T‐cell mediated immune response to a protein translated from the mRNA. They have been shown to exhibit a significant level of immunity.^8^ However, data regarding the safety of these vaccines are still lacking, and further studies are needed to uncover all the potential side effects.^9^ It is not uncommon to have neurological side effects following vaccination, as described in the literature.^10^ COVID‐19 vaccines have been linked to several CNS side effects as observed in trials, as well as they are being reported in case reports. The incidence is yet to be confirmed. As more and more people are being vaccinated, more side effects will be noticed.^11^ Side effects reported were weakness, numbness, ataxia, and more severe presentations such as encephalitis, myelitis, and demyelination with Guillain‐Barre syndrome.^12^ GBS development secondary to vaccines has been described before in literature.^13^ However, its development after the COVID‐19 vaccine is not well reported, and further studies are needed to expand on it. It was proposed that GBS development can occur due to molecular mimicry.^14^

Guillain‐Barre syndrome (GBS) is an acute immune‐mediated disease of peripheral nerves and nerve roots usually preceded by respiratory or gastrointestinal infection. It presents with progressive, ascending, symmetrical limb weakness and paresthesia with diminished or absent deep tendon reflexes, with or without respiratory and cranial nerves involvement.^15^ It is a rare condition (estimated 1–2 cases per 100,000 person‐years worldwide.^16^ In approximately two‐thirds of patients with GBS, an episode of acute infection precedes the neurological symptoms by 1–3 weeks.^17^ Infectious organisms recognized as potential triggers are diverse and include *Campylobacter jejuni*, cytomegalovirus, influenza viruses, herpes simplex virus (HSV), and human immunodeficiency virus (HIV).

Additionally, there is some evidence to suggest a temporal relationship between GBS and the receipt of various vaccines, including those for rabies, hepatitis (Hep) A and B, polio, and influenza.^18^ Such cases are infrequent, and the establishment of causality in most of these cases has proved controversial. However, a small increased risk of developing GBS, specifically after influenza vaccination, is relatively well established.^19^ Recently there have been multiple case reports of the temporal relationship between COVID‐19 vaccination and GBS development.

Our patient presented with bilateral upper extremity weakness and numbness four days following the vaccine. Initial evaluation revealed normal CSF studies and normal CBC, KFT, LFT. Nerve conduction studies were done, and they showed features of demyelinating disease. He was admitted to the hospital as a case of GBS. Following the diagnosis, he was started on Intravenous immunoglobulins for five days, which improved his symptoms on the following days. Which further confirmed the diagnosis. He continued to improve and was finally discharged with Follow‐ups. Upon reviewing in the clinic, he was completely fine, and his symptoms have completely resolved.

Patients with neurological findings should be enquired about recent vaccination history. It is of enormous importance, especially after the COVID‐19 mRNA vaccine, which is newly introduced, as it might link to the development of a wider variety of neurological presentations. The risks of the vaccines are not fully understood; however, the benefits of the vaccines appear to outweigh the risks that might be encountered.

## 4 CONCLUSION

COVID‐19 pandemic has burdened many countries and created many changes in the physical, social and economic aspects. COVID‐19 vaccines are an essential tool to help contain the pandemic. They have been proven to be very effective and safe, but at the same time, it is crucial to keep in mind the potential side effects of these vaccines. Patients who present with neurological complaints following the vaccine should be investigated for other causes first while considering that the vaccine can be a cause. As more patients are getting access to the coronavirus 2019 (COVID‐19) vaccines, neurologists are facing questions about neurological complications, benefits, and appropriate time of vaccination. GBS is one of the possible side effects that might be encountered after the COVID‐19 vaccine. If it occurs, it appears to be responsive to the treatment, and the benefits of administering the vaccine outweigh the risks. The frequency of its occurrence is still needed to be studied.

## CONFLICT OF INTEREST

The authors have no conflict of interest to declare.

## AUTHOR CONTRIBUTIONS

Ahmad S. Matarneh involved in history and physical examination, literature review, and manuscript writing. Alia Al Battah involved in history and physical examination, literature review, and manuscript writing. Khalid Farooqui involved in literature review and manuscript writing. Mohammed Ghamoodi involved in clinical care. Mohammed Alhatou involved in clinical care, manuscript writing, literature review, and mentor.

## ETHICAL APPROVAL

The case report was approved by Hamad Medical Corporation, MRC number MRC‐04‐21‐387.

## Data Availability

The data that support the findings of this study are available from the corresponding author upon reasonable request.
